# Circulating tumor DNA dynamics in advanced breast cancer treated with CDK4/6 inhibition and endocrine therapy

**DOI:** 10.1038/s41523-021-00218-8

**Published:** 2021-02-03

**Authors:** Olga Martínez-Sáez, Tomás Pascual, Fara Brasó-Maristany, Nuria Chic, Blanca González-Farré, Esther Sanfeliu, Adela Rodríguez, Débora Martínez, Patricia Galván, Anna Belén Rodríguez, Francesco Schettini, Benedetta Conte, Maria Vidal, Barbara Adamo, Antoni Martínez, Montserrat Muñoz, Reinaldo Moreno, Patricia Villagrasa, Fernando Salvador, Eva M. Ciruelos, Iris Faull, Justin I. Odegaard, Aleix Prat

**Affiliations:** 1 SOLTI Cancer Research Group, Barcelona, Spain; 2 Department of Medical Oncology, Hospital Clinic of Barcelona, Barcelona, Spain; 3 Translational Genomics and Targeted Therapies in Solid Tumors, August Pi i Sunyer Biomedical Research Institute (IDIBAPS), Barcelona, Spain; 4 Lineberger Comprehensive Cancer Center, University of North Carolina at Chapel Hill, Chapel Hill, NC USA; 5 Department of Pathology, Hospital Clinic of Barcelona, Barcelona, Spain; 6 Department of Clinical Medicine and Surgery, University of Naples Federico II, Naples, Italy; 7 Department of Medical Oncology U.O. Oncologia Medica 2, IRCCS Ospedale Policlinico San Martino, Genova, Italy; 8 Department of Medical Oncology, Hospital Universitario 12 de Octubre, Madrid, Spain; 9 Guardant Health, Inc., Redwood City, CA USA; 10 Department of Medicine, University of Barcelona, Barcelona, Spain

**Keywords:** Breast cancer, Predictive markers

## Abstract

Circulating tumor DNA (ctDNA) levels may predict response to anticancer drugs, including CDK4/6 inhibitors and endocrine therapy combinations (CDK4/6i+ET); however, critical questions remain unanswered such as which assay or statistical method to use. Here, we obtained paired plasma samples at baseline and week 4 in 45 consecutive patients with advanced breast cancer treated with CDK4/6i+ET. ctDNA was detected in 96% of cases using the 74-gene Guardant360 assay. A variant allele fraction ratio (VAFR) was calculated for each of the 79 detected mutations between both timepoints. Mean of all VAFRs (mVAFR) was computed for each patient. In our dataset, mVAFR was significantly associated with progression-free survival (PFS). Baseline VAF, on-treatment VAF or absolute changes in VAF were not associated with PFS, nor were CA-15.3 levels at baseline, week 4 or the CA-15.3 ratio. These findings demonstrate that ctDNA dynamics using a standardized multi-gene panel and a unique methodological approach predicts treatment outcome. Clinical trials in patients with an unfavorable ctDNA response are needed.

In hormone receptor positive (HR+)/HER2-negative advanced breast cancer (BC), CDK4/6i plus ET have remarkably improved survival outcomes and are now considered a standard treatment for most patients^1^. Although this is good news for patients suffering from metastatic BC, improving the efficacy of CDK4/6i and ET using novel treatment strategies might be challenging. On the one hand, no predictive biomarker exists to date to select patients who are going to progress early^2^. On the other hand, improving survival outcomes with new or additional therapies when the control arm has a median PFS of 25–27 months in the first-line setting will require huge personal, physical, and economic resources as well as long periods of follow-up^3–5^. This issue is not restricted to advanced BC but also other cancer types such as lung cancer.

Detection of ctDNA levels before and during therapy might improve CDK4/6i plus ET efficacy, stratify patients, and help design future trials with novel treatment strategies^6,7^. O´Leary and colleagues evaluated early ctDNA dynamics in patients with *PIK3CA*-mutated HR+/HER2-negative metastatic BC treated with palbociclib and fulvestrant in PALOMA-3 trial^8^. A multiplex digital PCR assay was used and hotspot *PIK3CA* mutations in exons 9 and 20 were evaluated in plasma^8^. *PIK3CA* mutation levels from baseline to day 15 of therapy were associated with PFS independently of the treatment received. However, only 22% of patients with HR+/HER2-negative advanced BC had detectable *PIK3CA* mutations in plasma. To circumvent this problem, others argue that individualized gene panels according to each patient´s tumor´s genetic profile should be prioritized^9^.

We hypothesized that a standardized plasma-based sequencing assay that analyzes multiple genes simultaneously at baseline and after 4 weeks (cycle 2 day 1 [C2D1]) of CDK4/6i plus ET can identify patients with HR+/HER2-negative advanced disease with different treatment outcomes. To accomplish this, we undertook a prospective study from May/2016 to June/2019 of 50 consecutive pre and postmenopausal patients with metastatic HR+/HER2-negative BC treated as per standard practice with CDK4/6i and ET (Fig. 1a). Plasma samples were sequenced using the standardized Guardant360 assay v2.11, which can identify single nucleotide variants (SNV) and indels from 74 genes (Fig. 1b)^10^. Among 50 patients, 2 patients (4%) had insufficient plasma volume, 2 patients (4%) had missing samples and 1 patient (2%) was treated in the adjuvant setting after resection of a supraclavicular lymph node and was excluded. Finally, 45 patients (90%) were evaluable of whom 43 (96%) had ctDNA detectable at some level. Of those 45, 31 (69%) had ctDNA-positive disease (i.e. highest VAF detected ≥VAF 0.4% at some timepoint) and 14 (31%) were considered to have ctDNA-low disease (i.e. highest VAF detected <0.4% or non-detected at both timepoints). Of the 31 ctDNA-positive patients, 30 had ctDNA-positive disease at baseline and 1 had ctDNA-low at baseline but ctDNA-positive at cycle 2 (Fig. 1c and Table 1). Mutations in 42 genes were identified at baseline and the 4 most frequent altered genes were *PIK3CA, ESR1, TP53*, and *ATM* (Fig. 1d); ≥1 mutation with ≥VAF 0.4% in any of these 4 genes was found in 24 patients (53.3%). We had available tissue samples collected from archival biopsies before treatment with CDK4/6i plus ET in 26 patients. As shown in previous studies, all intrinsic molecular subtypes were identified using the PAM50 subtype predictor, although Luminal A and B subtypes predominated (Fig. 1e)^11,12^.

**Fig. 1:**
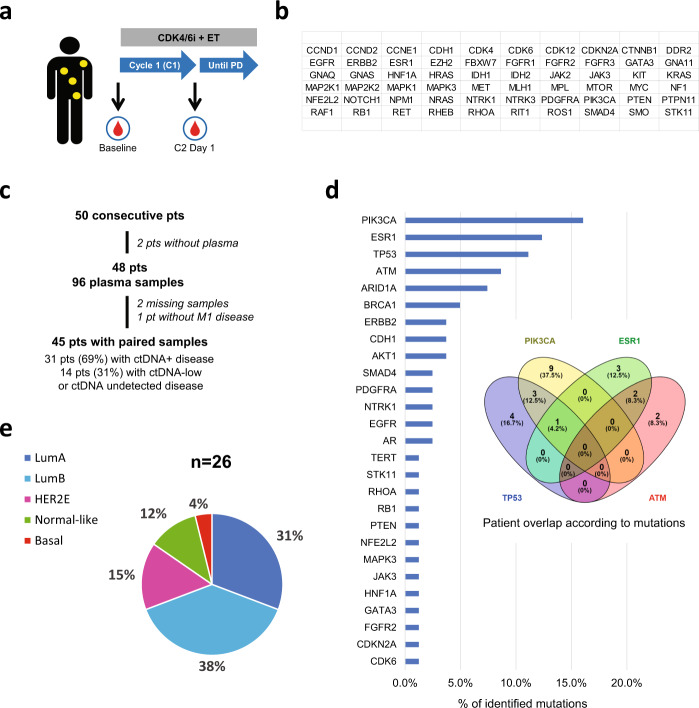
Description of the study. **a** Blood samples were extracted at baseline and after 1 cycle (i.e. 4 weeks) in patients with metastatic HR+/HER2-negative BC treated with CDK4/6i plus ET. **b** The list of 74 genes analyzed by Guardant360. **c** CONSORT diagram. **d** Frequency of gene mutations with ≥VAF 0.4% at baseline identified in the patient dataset and Venn diagram with the 4 most frequent mutations. **e** PAM50 distribution (LumA = Luminal A, LumB=Luminal B, HER2E = HER2-enriched). *PD Progressive disease, pts patients, M1 metastatic.

**Table 1:** Clinical features of the patient dataset according to ctDNA levels and dynamics.

	All patients *N* = 45	ctDNA-low *N* = 14	mVAFR-low *N* = 11	mVAFR-med/high *N* = 20
*Age*				
Median (range) yr	61.4 (39–87)	59.7 (39–87)	56.9 (45–72)	64 (42–75)
<65 yr—no. (%)	28 (62%)	8 (57%)	9 (82%)	11 (55%)
≥65 yr—no. (%)	17 (38%)	6 (43%)	2 (18%)	9 (45%)
*Line—no. (%)*				
First	21 (47%)	8 (57%)	5 (45%)	9 (45%)
Second	16 (36%)	5 (36%)	5 (45%)	5 (25%)
Third or more	8 (18%)	1 (7%)	1 (9%)	6 (30%)
*ECOG-PS*^*a*^*—no. (%)*				
0	17 (38%)	5 (36%)	5 (45%)	7 (35%)
1	27 (60%)	9 (64%)	6 (55%)	12 (60%)
2	1 (2%)	0	0	1 (5%)
*Endocrine therapy—no. (%)*				
Aromatase inhibitor	15 (33%)	4 (29%)	3 (27%)	8 (40%)
Fulvestrant	26 (58%)	9 (64%)	7 (64%)	10 (50%)
Tamoxifen	4 (9%)	1 (7%)	1 (9%)	1 (5%)
*Type of CDK4/6 inhibitor—no. (%)*				
Palbociclib	40 (89%)	12 (86%)	10 (91%)	18 (90%)
Ribociclib	5 (11%)	2 (14%)	1 (9%)	2 (10%)
*Disease site—no. (%)*				
Visceral	2 (64%)	10 (71%)	7 (64%)	12 (60%)
Non visceral	16 (36%)	4 (29%)	4 (36%)	8 (40%)
Bone-only	10 (22%)	1 (7%)	4 (36%)	5 (25%)
*Number of metastatic locations—no. (%)*				
<3	23 (51%)	8 (57%)	7 (64%)	8 (40%)
≥3	22 (49%)	6 (43%)	4 (36%)	12 (60%)
*“De novo” metastasis—no. (%)*				
No	33 (73%)	10 (71%)	7 (64%)	15 (75%)
Yes	12 (27%)	4 (29%)	4 (36%)	5 (25%)
*Prior hormone sensitivity—no. (%)*				
Sensitivity	31 (69%)	9 (64%)	7 (64%)	15 (75%)
Resistance^b^	14 (31%)	5 (36%)	4 (36%)	5 (25%)
*Histology—no. (%)*				
Ductal	32 (71%)	11 (79%)	8 (73%)	13 (65%)
Lobular	9 (20%)	3 (21%)	3 (27%)	3 (15%)
Other	4 (9%)	0	0	4 (20%)
*Grade—no. (%)*				
1	5 (11%)	2 (14%)	2 (18%)	1 (5%)
2	19 (42%)	5 (36%)	6 (55%)	8 (40%)
3	12 (27%)	5 (36%)	2 (18%)	5 (25%)
Unknown	9 (20%)	2 (14%)	1 (9%)	6 (30%)
*Ki67 (%)—no. (%)*				
1–14	9 (20%)	4 (29%)	2 (18%)	3 (15%)
15–20	3 (7%)	1 (7%)	0	2 (10%)
>20	25 (56%)	7 (50%)	6 (55%)	12 (60%)
Unknown	8 (18%)	2 (14%)	3 (27%)	3 (15%)

^a^*PS*: performance status.

^b^Hormone resistance defined as relapse while on the first 2 years of adjuvant ET, or progression of disease within first 6 months of first-line ET for advanced BC, while on ET.

A total of 159 mutations (SNV and indels) were found at baseline; of them, 93 were detected in C2D1. From the 159 mutations in baseline, 79 had a VAF ≥ 0.4% in 31 patients (mean of 2.6 alterations per patient), 60 of these mutations were detected at some level in C2D1. 31 mutations were detected in C2D1 and not in baseline, but only 3 mutations with VAF ≥ 0.4% were detected at C2D1 and not in baseline (Supplementary Fig. 3 and Fig. 4). Mean VAF (mVAF) of the 79 mutations was 6.2 at baseline and 5.1 at C2D1 (*p*-value=0.040) (Fig. 2a). Any decrease in VAF at C2D1 compared to baseline was observed in 71% (56/79) of the tracked mutations. To capture the magnitude of ctDNA response, a VAFR from C2D1 to baseline was calculated for each genetic mutation and a mVAFR was computed for each patient (Fig. 2b and Methods). 35% of patients had mVAFR of ≤0.3 (mVAFR-low), 29% had mVAFR of 0.31–0.99 (mVAFR-medium) and 35% had a mVAFR of ≥1.0 (mVAFR-high) (Fig. 2c). Finally, no clinical features were found specific of a particular ctDNA group (Table 1).

**Fig. 2:**
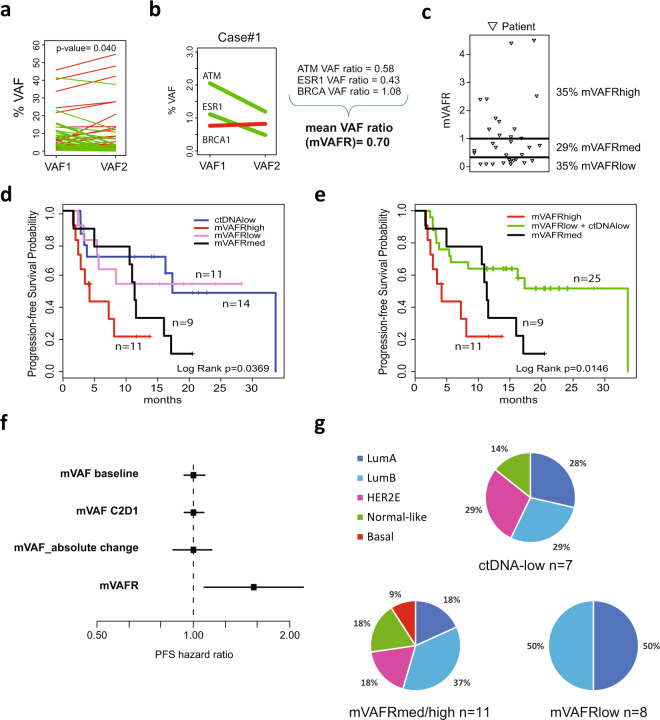
ctDNA dynamics and survival outcome. **a** VAF changes of 79 detected mutations from baseline to C2D1. *p*-value was calculated with Wilcoxon rank sum test. **b** Illustration of a case and its mVAFR. **c** Distribution plot of patients with mVAFR-low (mVAFR of ≤0.3), mVAFR-medium [mVAFRmed] (mVAFR of 0.31–0.99) and mVAFR-high (mVAFR of ≥1.0). **d** PFS based on ctDNA dynamics. **e** PFS based on ctDNA dynamics after combining the VAFR-low and ctDNA-low groups. **f** PFS hazard ratio forest plots across 4 different methods of assessing mVAF as a continuous variable: mVAF baseline, mVAF at C2D1, absolute change of mVAF between baseline and C2D1 and mVAFR of all mutations with a VAF ≥ 0.4 at baseline or C2D1. **g** Distribution of the intrinsic subtypes in the mVAFR-low group (*n* = 4 Luminal A [LumA], *n* = 4 Luminal B [LumB], *n* = 3 non-available tissue [NA]), mVAFR-medium/high group (*n* = 2 LumA, *n* = 4 LumB, *n* = 2 HER2-Enriched [HER2E], *n* = 2 Normal-like, *n* = 1 Basal-like, *n* = 9 NA) and ctDNA-low group (*n* = 2 LumA, *n* = 2 LumB, *n* = 2 HER2E, *n* = 2 Normal-like, *n* = 7 NA).

With a median follow-up of 20.4 months, a significant association between the various ctDNA groups and PFS was observed across all patients (*p*-value=0.02) (Fig. 2d, e). Compared to the mVAFR-high group, the mVAFR-low group was associated with better PFS (not reached (NR) (95% confidence interval [CI] 5.40-NR) vs. 4.2 months (95% CI 2.41–11.60); adjusted hazard ratio [aHR]=0.31, 95% CI 0.09–1, *p*-value=0.049). Similarly, the mVAFR-low and ctDNA-low groups combined was associated with better PFS compared to the mVAFR-high group (33.7 (95% CI 5.3–33.7) vs. 4.2 months; aHR=0.25, 95% CI 0.09–0.7, *p*-value=0.008). The results in ctDNA-low group are in line with what was previously described in other metastatic tumors, as low ctDNA levels seem to be a good prognostic feature. In addition, mVAFR as a continuous variable was also found significantly associated with PFS (aHR per 1-unit increase=2.07, 95% CI 1.2–3.5, *p*-value=0.008) (Supplementary Table 2). Of note, mVAF at baseline, or mVAF at C2D1 or absolute changes in mVAF (delta-VAF) were not found associated with PFS when evaluated as continuous variables (Fig. 2f and Supplementary Table 1). These results are in consonance with different studies that assessed ctDNA dynamics and have showed that higher pretreatment VAF acts as poorer prognostic factor but has no predictive value^13,14^. On the other hand, there is not a standard method yet to assess ctDNA dynamics; several studies have used the delta-VAF or a ratio between the two timepoints^8,13,15–17^. However, while delta-VAF is able to stratify patients similarly to on-treatment VAF, it only assesses absolute VAF changes and equates patients with low VAF who had a significant decrease in ctDNA level and patients with a higher VAF and smaller decrease in ctDNA level. Interestingly, no patient with non-luminal tumors was identified as high ctDNA responder (i.e. mVAFR-low), consistent with previous reports in advanced HR+/HER2-negative disease associating the Luminal phenotype with better prognosis and response to ET compared to non-luminal tumors (Fig. 2g)^18,19^.

At baseline, median CA-15.3 value was 45 U/mL (7–6,672), and 26 patients (61%) had high CA-15.3 values (>35 U/mL). At C2D1, median CA-15.3 value was 42 U/mL (range 8–9,868), and 19 patients (51%) had high CA-15.3 values. No significant differences in CA-15.3 levels were observed between baseline and C2D1 (*p*-value=0.350). The median ratio of CA-15.3 between C2D1 and baseline was 1.05 (range 0.4–1.6). No correlation was observed between CA-15.3 ratio and ctDNA mVAFR (correlation coefficient = −0.021). The levels of CA-15.3 at baseline or C2D1, and the CA-15.3 ratio, were not found associated with PFS (data not shown).

Our study has limitations worth noting. First, the limited sample size, which precludes more in-depth analysis within subgroups of patients. For example, identification of an optimal mVAFR cutoff to define prognosis will require a larger sample set. Second, there is not yet a standardized method to assess the ctDNA dynamics. We are aware that the arithmetic mean of VAFRs could result in overestimation of the average ctDNA change due to the nonadditive nature of the ratios and the small number of detected mutations per patient. To address this, we assigned fixed VAFR values of 10 or 0.1 (Methods). As this mitigation strategy could not cover all potential cases, we have studied other possibility taking the logarithm of each ratio, and then taking the mean of logarithms. The correlation coefficient between both scores (i.e. simple arithmetic mean ratio and logarithm mean ratio) was 0.94 and the Kappa concordance score between mVAFR-based groups of patients and mVAFR_log-based groups of patients, 0.88. As expected, this scoring method was also found associated with PFS (Supplementary Section). The best method to assess ctDNA dynamics should be determined in future studies. Third, the short follow-up time does not allow associations with overall survival. Fourth, we did not explore if tracking ctDNA levels of particular genes is better than tracking any detected altered gene in plasma. Nonetheless, a strong argument in favor of our approach is that it does not rely on specific genes but rather on the dynamic changes of the altered genes identified before initiating treatment. Fifth, it is unclear if this approach and methods will be applicable for other therapies or other cancer types. Lastly, we cannot completely exclude that some of the alterations identified are from clonal hematopoyesis (CH). However, we try to correct for clinical CH in two ways. First, we excluded any variant present at <0.4% since the vast majority of CH variants are present below this level and this excludes them from contributing to the assessment. Second, our method averages all variants present, which dilutes the impact of any atypical CH variant that may have exceeded the VAF threshold. In such cases, the CH variant would remain unaffected by therapy and remain at a static VAF and, as such, would not contribute to any changes in tumor ctDNA fraction, although it could dilute somewhat any changes observed in true tumor ctDNA. Finally, our findings will require further validation in patients with advanced BC treated with CDK4/6i plus ET.

Our findings have several potential clinical implications. Most importantly, they suggest that early ctDNA dynamics using a multi-gene assay and a particular statistical methodology serve as a general biomarker to identify patients with advanced BC who are at high risk of progression during standard therapy with CDK4/6i and ET, giving the opportunity to intervene and change the treatment or add another treatment early. Notably, the biomarker seems independent of baseline clinical features, tumor marker CA-15.3 and the clinical setting, and the relationship of low ctDNA-responders with the non-luminal subtypes is weak. It will be important to also assess its value on the outcome of additional therapies and cancer types. Overall, our findings support the notion that monitoring ctDNA should be an integral part during drug development and should allow the design of novel clinical trials in key patient populations, such as those patients with an unfavorable ctDNA response.

## Methods

### Study design and patients

This is a prospective, single-center study in 50 consecutive patients with advanced BC. Eligible patients were ≥18 years of age with histologically confirmed HR+/HER2-negative inoperable or metastatic BC treated with a CDK4/6i and ET. Blood samples for sample collection were obtained at baseline and at C2D1. Clinical data, results of computed tomography (CT) imaging, and serial blood samples were collected as per standard practice. The study was performed in accordance with Good Clinical Practice guidelines and the World Medical Association Declaration of Helsinki. The study was approved by the local institutional research ethics committee, and all patients provided written informed consent.

### Plasma samples

Approximately 30 mL of venous blood was extracted at each timepoint and collected in EDTA tubes. Blood was processed within 2 h after the collection. Centrifugation at 1600*g* for 10 minutes at 4 °C was performed to separate the plasma from the peripheral-blood cells. We obtained approximately 12 mL of plasma per patient and timepoint, and plasma was immediately aliquoted in 1.5 mL tubes and then we centrifugated them at 16,000*g* at 4 °C for another 10 minutes to remove the residual supernatant and any remaining contaminants including cells. Separated plasma was aliquoted in a 1.5 mL tube and immediately stored in a deep freezer at −80 °C.

Cell-free DNA (cfDNA) was extracted from 1.5 ml aliquots of plasma using the QIAamp circulating nucleic acid kit (Qiagen), concentrated using Agencourt Ampure XP beads (Beckman Coulter), and quantified by Qubit fluorometer (Life Technologies, Carlsbad, CA, USA). All cfDNA isolation and sequencing was performed at Guardant Health (Redwood City, CA, USA).

### DNA sequencing

Genomic alterations (SNV, insertions and deletions (indels) and amplifications) were detected from cfDNA extracted from plasma samples using a broad targeted NGS-based 74-gene panel (Guardant360), including coverage of the most prevalent tumor suppressor genes in human cancers (Fig. 1b). After isolation of cfDNA by hybrid capture, the assay was performed using molecular barcoding and proprietary bioinformatics algorithms with massively parallel sequencing on an Illumina Hi-Seq 2500 platform in a CLIA/CAP accredited laboratory (Guardant Health; Redwood City, CA, USA).

### ctDNA response definition

We filtered somatic mutations with VAF ≥ 0.4% either at baseline (C1D1) or C2D1, based on 95%–100% limits of detection for this technology. Those patients with VAF < 0.4% at both timepoints were considered low-shedding tumors. We calculated the proportional change for all variants detected between the 2 timepoints (VAF ratio [VAFR] = VAF_C2D1/VAF_C1D1). For variants detected at 1 timepoint but not the other, VAF was set to 0. We considered all undetected variants at C2D1, or variants with a VAF < 0.4% at C2D1, to have a VAFR of 0.1 as minimum. The reason is to prevent skewing of the average by variance introduced by quantitation variability below 0.4% VAF. We considered all new variants detected at C2D1 but not at baseline to have a VAFR of 10 as maximum. The reason is to prevent skewing of the average by variance introduced by quantitation variability below 0.4% and by dividing by numbers that approach zero. Finally, a mVAFR was calculated per patient taking the average of all VAFR.

### CA-15.3 determination

The CA-15.3 assay was performed by the BRAHMS Kryptor Plus compact controller using TRACE (Time-Resolved Amplified Cryptate Emission) technology. CA-15.3 was considered elevated when it was above the normal upper limit (35 U/mL).

### PAM50 subtype determination

A minimum of ∼125 ng of total RNA from formalin-fixed paraffin embedded tumor samples was used to measure the expression of the 50 PAM50 subtype predictor genes and 5 housekeeping genes using the nCounter platform (Nanostring Technologies, Seattle, USA).

### Response evaluation by image

CT scan and bone scintigraphy were performed as per standard practice. RECIST 1.1 was used to evaluate tumor responses.

### Statistical analysis

The primary objective was to evaluate the association of ctDNA dynamics from baseline to C2D1 and PFS. PFS was defined as the time from initiation of treatment until progression or death. Univariate and multivariate Cox proportional hazard regression analysis was used to investigate the association of each variable with PFS. VAF changes between timepoints were calculated with Wilcoxon Rank Sum Test. The significance level was set to a 2-sided alpha of 0.05. *p*-value was calculated with Wilcoxon Rank Sum Test. All analyses were performed with R code 3.6.

### Reporting summary

Further information on research design is available in the Nature Research Reporting Summary linked to this article.

## Supplementary information

Supplementary material

Data set

Reporting Summary Checklist

## Data Availability

The data generated and analyzed during this study are described in the following data record: 10.6084/m9.figshare.13365521^20^. The ctDNA and clinical data are available in two separate tabs in the Excel spreadsheet “ctDNA and clinical dataset.xlsx”, which is openly available and shared as part of the figshare data record^20^. The ´CDK series - ctDNA and clinicopathological dataset´ is not publicly available in order to protect patient privacy. Requests for access to this dataset can be made to the corresponding author.
