# Supplementary material for: Circulating tumor DNA dynamics in advanced breast cancer treated with CDK4/6 inhibition and endocrine therapy
Source: NPJ Breast Cancer. 2021 Feb 3;7:8. doi: 10.1038/s41523-021-00218-8 (PMC7859394; doi:10.1038/s41523-021-00218-8)
Supplement: Supplementary file 1 — Supplementary material [file 41523_2021_218_MOESM1_ESM.pdf]

Supplementary material

Logarithm transformation of mVAFR_log

The mVAFR_log was defined as the mean of the logarithm of all VAF ratios (VAF_{post}/VAF_{pre}) for all mutations in each time point:

$$(1)\ \boldsymbol{mVAFR\_log} = \boldsymbol{m}(\log\left(\frac{VAF_{post}}{VAF_{pre}}\right))$$

We divided the population with ctDNA-positive in tertiles according to the mVAFR_log.

The group of patients with ctDNA low was consider again as an independent group. We consider then 4 groups, mVAFR_log-low (first tertile), mVAFR_log-medium (second tertile) and mVAFR_log-high (third tertile). Both mVAFR_log as a continuous variable (HR=2.85, CI 95% 1.00-8.20, p-value=0.05) and m VAFR_log groups (HR=3.99 for mVARF_log-high group compared to mVARF_log-low, CI 95% 1.24- 12.81, p-value=0.02) were associated to PFS.

Spearman correlation between mVAFR and mVAFR_log was 0.94 (figure 1).

Figure 1. Spearman correlation between mVAFR and mVAFR_log

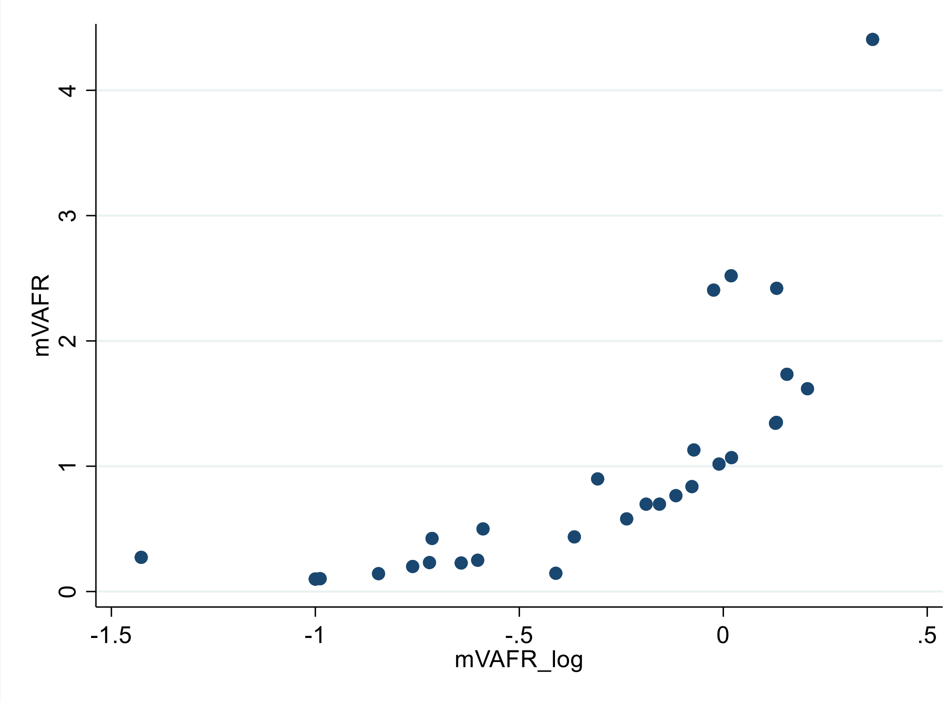

The Kappa concordance score between mVAFR-based groups and mVAFR_log-based groups was 0.88.

Figure 2. Single nucleotide variants (SNV), indels and copy number variations (CNV) found at baseline

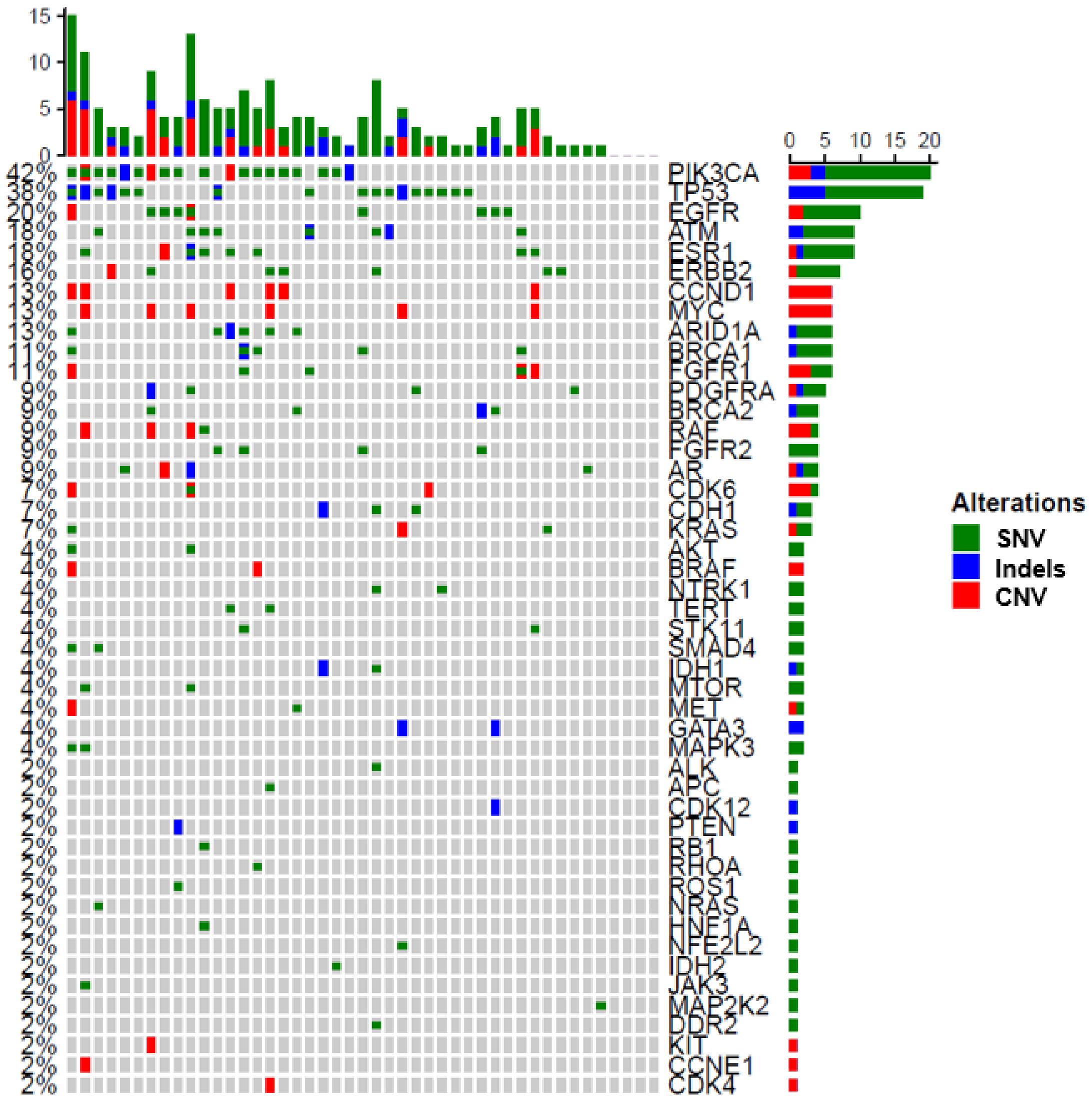

Table 1: Univariate Cox regression analysis of progression free survival

	HR	95% CI	p-value
mVAF baseline	1.00	0.93-1.08	0.94
mVAF C2D1	1.01	0.95-1.07	0.77
mVAF_absolute change	1.03	0.90-1.20	0.62
mVAFR	1.57	1.10-2.23	0.012

Table 2. Multivariable Cox regression analysis of progression free survival considering mVAFR as continuous variable

	HR	95% CI	p-value
mVAFR	2.07	1.21-3.54	0.08
Visceral disease	0.28	0.04-1.85	0.19
≥3 metastatic locations	0.53	0.07-3.68	0.52
Hormone-resistance	3.57	0.84-15.18	0.09
Line of treatment			
Second	1.26	0.31-4.69	0.79
Third or more	6.86	1.24-38.02	0.03
Endocrine therapy			
Fulvestrant[1]	1.23	0.36-4.26	0.74
Tamoxifen[1]	2.54	0.25-25.45	0.43

[1] HR with respect to aromatase inhibitor

Table 3. Multivariable Cox regression analysis of progression free survival considering mVAFR by groups

	**HR**	**95% CI**	**p-value**
mVAFR groups			
mVAFR-med[1]	2.75	0.85-8.89	0.09
mVAFR-high[1]	3.58	1.26-10.24	0.02
Visceral disease	0.49	0.15-1.60	0.24
≥3 metastatic locations	1.15	0.36-3.71	0.82
Hormone-resistance	3.22	1.10-8.81	0.03
Line of treatment			
Second	1.26	0.38-2.85	0.64
Third or more	2.07	0.53-8.10	0.30
Endocrine therapy			
Fulvestrant[2]	1.04	0.38-2.85	0.95
Tamoxifen[2]	0.82	0.08-7.99	0.86

[1] HR with respect to low-ctDNA and mVAFR low group (reference category).

[2] HR with respect to aromatase inhibitor

Figure 3. SNV and indels found at baseline

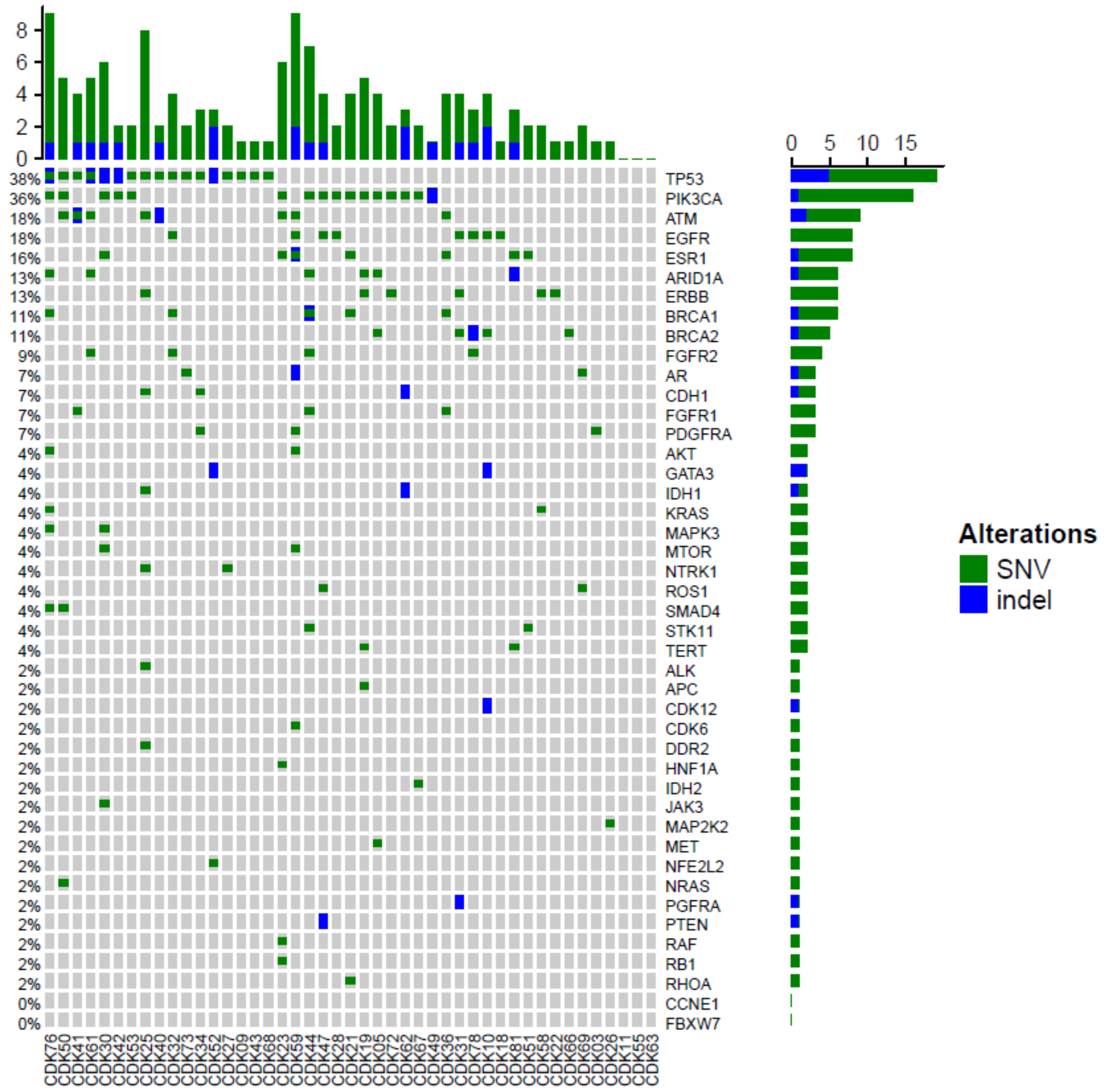

Figure 4. SNV and indels found at C2D1

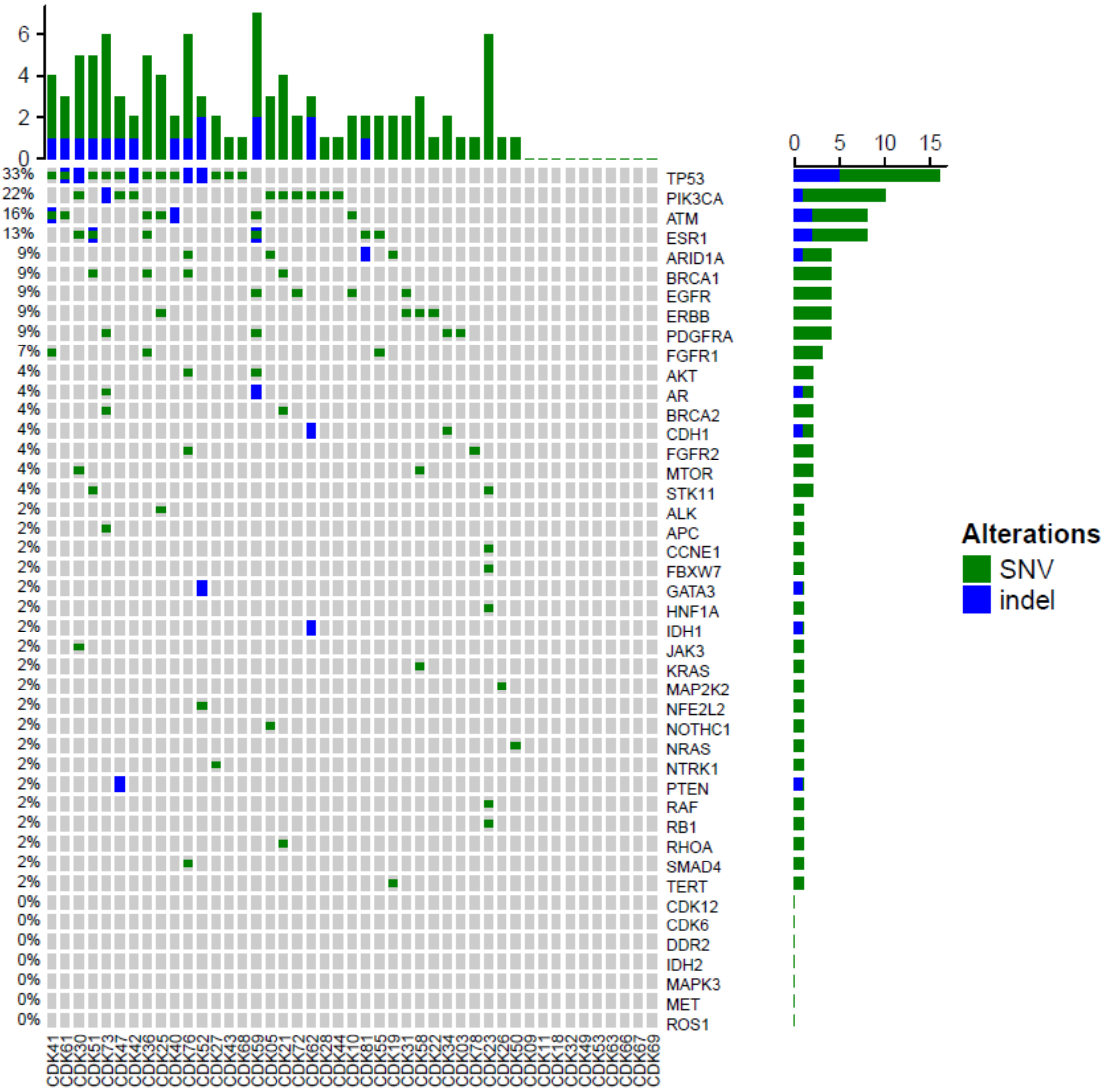

ctDNA and clinical dataset. The ctDNA and clinical data analyzed during this study are described in two separate tabs in the Excel spreadsheet 'ctDNA and clinical dataset.xlsx', which is openly available and shared as part of the figshare data record in the following data record: https://doi.org/10.6084/m9.figshare.13365521[20].
